# The Resilience Program: preliminary evaluation of a mentalization-based education program

**DOI:** 10.3389/fpsyg.2015.00753

**Published:** 2015-06-16

**Authors:** Poul L. Bak, Nick Midgley, Jin L. Zhu, Karen Wistoft, Carsten Obel

**Affiliations:** ^1^The Danish Committee for Health Education, CopenhagenDenmark; ^2^Anna Freud Centre and Research Department of Clinical, Educational and Health Psychology, University College London, LondonUK; ^3^Institute of Public Health, Aarhus University, AarhusDenmark; ^4^Department of Education, University of Greenland, NuukGreenland

**Keywords:** mental health education, mentalization, theory of mind, resilience, conflict prevention

## Abstract

In order to manage with the burden of mental health problems in the world we need to develop cost-effective and safe preventive interventions. Education about resilience to support the ability to cope with life challenges in general, may be a useful strategy. We consider the concepts of Theory of Mind and Mentalization to be relevant in this context. In this paper we describe a simple modular intervention program based on these concepts which can be tailored to specific needs and situations in individual therapy as well as group levels. The program has shown promising results in pilot studies and is now tested in controlled trials in settings such as schools and educational institutions, adults diagnosed with ADHD, and children in care.

## Introduction

There is an increasing awareness that the burden of mental health problems in the world cannot be addressed by therapeutic interventions alone (Kazdin and Blase, 2011). Alongside improving treatments, we need to develop cost-effective and safe preventive interventions (Fonagy et al., 2005; Rhule, 2005; Roth and Fonagy, 2006; O’Connell et al., 2009; Kazdin and Blase, 2011).

One promising approach is structured mental health education. A number of meta-analyses suggests that mental health education has positive effects on perceived health and behavior in a wide range of settings (Montgomery et al., 2006; Knouse et al., 2008; Donker et al., 2009; Baskin et al., 2010; Xia et al., 2011), such as parent management training (Montgomery et al., 2006), anxiety (Hedman et al., 2011), eating disorders (Perkins et al., 2009), and in pediatric health care (Cushing and Steele, 2010). The apparent most important behavioral components in these programs are specific goal setting, self-monitoring, feedback, and contingency management (Cushing and Steele, 2010), but it is not always clear whether these programs also build a capacity for increased resilience to withstand future challenges.

### Resilience and Mentalization

Resilience is defined as successful adaptation to adversity, including successful recovery from adverse life events and sustainability in relation to life challenges, individually and on group- and community-levels (Zautra et al., 2010).

The term Mentalization refers to the skills involved in understanding mental states, not only in others but also one’s own mental states as well as their connections with behavior. This is central in mutual understanding of relationships, self-control, motivation, and flexible understanding of what is going on in the world around. Theory of Mind is thus an integrated part of mentalization (Fonagy et al., 2002; Fonagy and Bateman, 2011; Liotti and Gilbert, 2011).

A compromised ability to mentalize is considered as a core neuropsychological deficit in autism spectrum disorders (Castelli et al., 2002; Philip et al., 2012) and borderline personality disorder (Allen and Fonagy, 2006). Individuals with psychiatric disorders such as schizophrenia, obsessive-compulsive personality disorder, psychosomatic disorders, eating disorders, panic disorders, and depression may also be in a non-mentalizing state of mind. (Hains and Arnsten, 2008; Fonagy and Bateman, 2011; Sharp and Venta, 2012). The same holds for completely normal individuals in severe distress.

The psychological research about mentalization is supported by neuro-imaging studies demonstrating frontal and temporal functional changes (Fonagy et al., 2005; Bisson, 2007; Blakemore, 2008; Hains and Arnsten, 2008; Lombardo et al., 2009; Fonagy and Bateman, 2011; Gweon et al., 2012; Zaki and Ochsner, 2012; Nolte et al., 2013; Happé and Frith, 2014). Mentalization based treatment programs have proved valuable in the treatment of adults with borderline personality disorder (Bateman and Fonagy, 2013), as well as in work with adolescents who self-harm (Rossouw and Fonagy, 2012). These results have stimulated interest in extending mentalization knowledge and tools from adult psychiatry in to child and adolescent psychiatry and in to mental health promotion in coping with stressful challenges (Midgley and Vrouva, 2012). For example, trials have indicated that a mentalization-based approach can be effective in reducing bullying in schools, when applied at a whole-system level (Fonagy et al., 2009).

Based on these findings we have developed a modular mentalization-based intervention program that we call ‘the Resilience Program’ in which a social field education model is combined with a self-directed web-based approach. The aim of this article is to describe the Resilience Program briefly and present preliminary results and ongoing studies.

### The Resilience Program

The Resilience Program is a flexible web-based modular mental health education program that can be used in general mental health promotion as well as in supporting people with mental health problems independent of character and complexity. The program can be used in any organizational context (e.g., youth work, education, social care) and can be integrated with any daily routines and in combination with other interventions, with low or high intensity. Hitherto our pilot experiences clearly indicate that following a brief period of training the program can be used by any professional and by lay persons, including parents, and students.

The Resilience Program website^1^ contains all information and a number of presentations about the Program, which consists of knowledge about resilience, mentalization and self-control, social learning theory, cognitive training, and neuroscience. This knowledge is transformed into a coherent yet simple, easy to understand set of presentations combining daily language texts, pictures, and short films.

It is possible to use the Resilience Program as a completely self-directed program. However, the program is most often introduced to target groups in short lectures and courses followed by discussions, group work, and follow-up supervision. Whenever possible we use a social field model of delivery, for instance a whole school intervention approach including both teachers and parents. Afterward teachers and parents use whichever program modules they find relevant to their setting, in talks and education with their children (down to the age of 6–7 years) and adolescents. The program is thus organized as free-standing modules that can be combined for individual purposes. It is clear from the material what is applicable for children and adolescents and what is useful for the adults around them.

For illustrative purposes, we present two examples from the Resilience Program (copied directly from the program website) describing in daily language and metaphors what is going on in the brain and our minds in mentalizing and non-mentalizing states.

The Story of the House of Thoughts is a metaphor for the core idea about mentalizing and non-mentalizing states. It is read aloud by parent, teacher, or instructor. Children can then eventually write or draw their own personal version of the story – their own house.

#### The Story of the House of Thoughts

In some way, we may say that our thoughts live inside our heads. Imagine that your thoughts live in a house with many rooms where you can wander around and discover them. When you discover thoughts, you are using the world’s finest tool – your attention, which is a kind of spotlight. When you throw light on a thought, you spot it and discover it. Thereafter you can shift your attention and discover another thought.

The House of Thoughts has plenty of rooms – a number of exciting thoughts may live in one room, perhaps some sad or angry thoughts live in another room and various happy thoughts live in a third room.

From The House of Thoughts, your thoughts can call you if they want to be discovered. This may be really exciting and good, but could be irritating too – especially if the thoughts are annoying and keep knocking all the time, trying to take charge over your attention. In the case where you have sad, anxious, or angry thoughts that take charge and force you into their room all the time, you might end up believing there are no exciting or happy thoughts to be found anywhere and that is not much fun.

… Yet this is not the case at all. All the happy and exciting thoughts are just waiting in other rooms in the House of Thoughts, waiting for you to discover them with your attention. Maybe there are even tools to be found in one room that could be used to fix some other thoughts in another room in the house. There may also be thoughts in a room that need to be left in peace, so they will not disturb you too much. If you often go to explore The House of Thoughts with your attention, then it becomes easier to be in charge of your thoughts.

**FIGURE 1:**
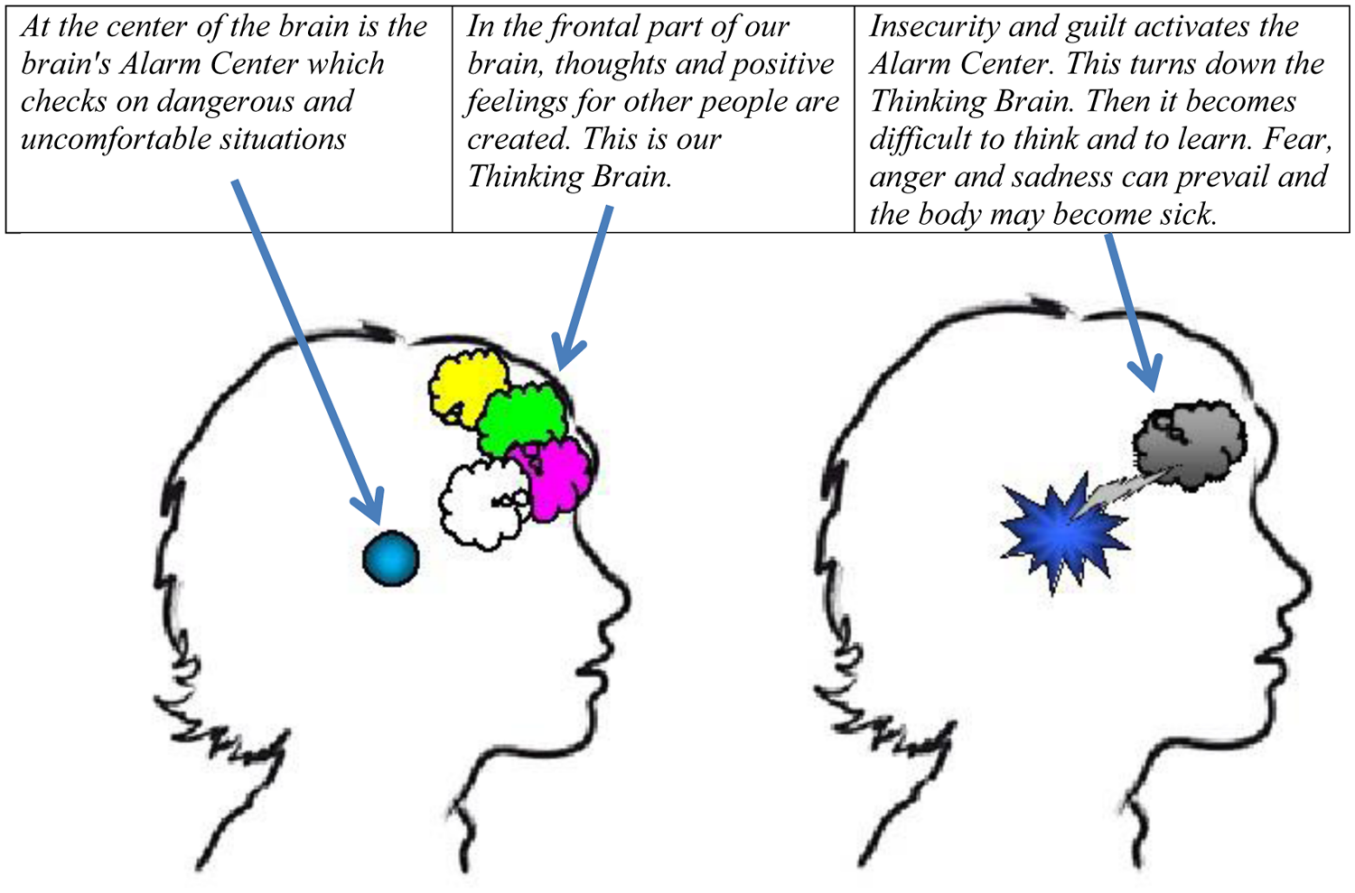
**Picture from the Resilience Program describing “the Thinking Brain” and the “Alarm Center”**.

The section about the Thinking Brain and the Alarm Center explains the neurobiology behind mentalizing in simple terms (see **Figure 1** and the italic text below the figure):

#### The Thinking Brain and the Alarm Center

Here, you can read about how your brain works when all is well and when things go wrong.

Unpleasant and dangerous situations can cause the alarm center to be over-sensitive. This means that the next time you are in a situation that resembles the ‘danger’ situation, the center may overreact with the result that you become afraid, angry, or sad – perhaps without any reason at all. It becomes difficult to think rationally – instead you react instinctively to ‘survive’ mentally and socially.

It is obviously good that the alarm center takes over when we are facing real danger. If your life is at risk there is no time to consider the pros or cons of taking action – you have to react promptly with fight or flight. However, it is not so desirable if the thinking brain turns off when there is no serious danger to you. An example is when you go blank in an exam situation, or when you panic about something that in fact is not dangerous at all. If your alarm center has been over-sensitive, it can be provoked just by thinking about an unpleasant situation.

The only thing you learn when you are alarmed is to be on guard in similar situations. You do not become more resilient, but you are at risk of becoming more vulnerable. Thoughts about your psychological and social survival will dominate your thinking. Vulnerability can be seen as anger, fear, and sadness.

If you on the other hand, become overprotected and do not face any challenges, your alarm center will believe that everything is ‘dangerous,’ which makes you vulnerable as well.

Very unpleasant and dangerous situations (traumas, accidents, and assaults) of course increase the risk of over-sensitizing the alarm center. Unfortunate micro-events can, by chance, also create permanent over-sensitivity in the alarm system (e.g., a horror movie). The most frequent cause of imbalance in the alarm center is insecurity in everyday life for example within family, in school or at work, and stress at a level that overloads the working memory and causes loss of overview.

Other people’s thoughts are invisible. That is why we sometimes misunderstand another person and believes that he or she does not want any good for us. Such thoughts can trigger the alarm center. If the other person is in an alarm state as well, we have two alarmed brains fighting each other and/or fleeing from each other.

Fortunately, the brain can be trained to become resilient instead of becoming vulnerable. When the thinking brain and the alarm center face appropriate challenges, neither to big nor too small, the thinking brain is able to control the alarm center, so it is not triggered without reason. The brain’s working memory is trainable too, thus making it easier to cope with life.

### Evaluation of the Resilience Program

The Resilience Program has been developed in 2005–2007 inspired by mentalization research, cognitive, and neuroscience and social learning theory. The program was pilot tested in the Municipality of Aarhus in Denmark in 2008–2010 (Lundgaard Bak, 2012). The primary result was that the program had a very high feasibility.

In 2013 we began to investigate the efficacy and efficiency of the program, using the intervention methods described above, in four controlled studies: a school study involving 60 schools and a youth education study involving 16 educational institutions; a study with 9,000 looked after children; and a study with 8,000 young people with ADHD. Data collection will start in late 2015 and be repeated in the following years. Results will be presented in 2016–2018. Trial protocols can be seen at the program website^2^ on the sub-site ‘about us.’

The Resilience Program is currently implemented locally in five European countries and is also being tested in studies with various other methodologies by independent researchers.

## Materials and Methods

In order to illustrate the potential use of the Resilience Program, we here present 3 years follow up results from an exploratory pilot study in spring 2011 using the first version of the Resilience Program in a low-income city area in Denmark.

### Pilot Study Background Information

Ninety percentage of the population in the target area of the study is immigrants from Middle East countries. In the area, there are several social clubs for adolescents. In 2009–2011, one of the clubs was challenged by increasingly severe disruptive behavior among the adolescents, for reasons which the managers of the clubs were not able to clearly identify. For that reason the managers asked for this intervention. At the time of intervention in spring 2011, 130 adolescents were registered members of the club.

The staff in the Municipality of Aarhus are regularly offered post-graduate education. In the period 2009–2011, the staff in the trial club in the area had two other post-graduate courses, one about coaching, and one about body relaxation.

Because of encouraging findings from the pilot trial described here, the managers implemented the same program and training in a neighbor club in the area in the late 2012. The neighbor club did not receive the other mentioned post-graduate courses. Data before and after intervention from this neighbor club is included in the study.

### Intervention Method

All staff members received a 3-days Resilience Program course with follow-up supervision for 3 months. The staff introduced the adolescents to the program. A sub-group among the adolescents received a more intensive education (45 min × 6).

### Data Collection

The results in the social club study are based on the following data:

• The frequency of incidents where staff members use physical force in high-risk conflicts in order to protect persons from physically damaging themselves or other persons. We have incident data from the trial club and the neighbor club in the period 2009–2014.
• Staff sick leave. This is standard administrative data in the organization. We have data from all clubs in the municipality from 2008 to 2014.
• Questionnaire data. In spring 2014 the staff in the trial club and the neighbor club filled out a questionnaire asking them to evaluate how meaningful (on a 10 point scale) they *presently* consider their post-graduate courses from 2011: the coach course, the body relax course and the Resilience Program course. They were also asked if they specifically use Resilience Program modules in direct talks and education with adolescent in their present daily work in the club.

## Results

### Force Incidents

**FIGURE 2:**
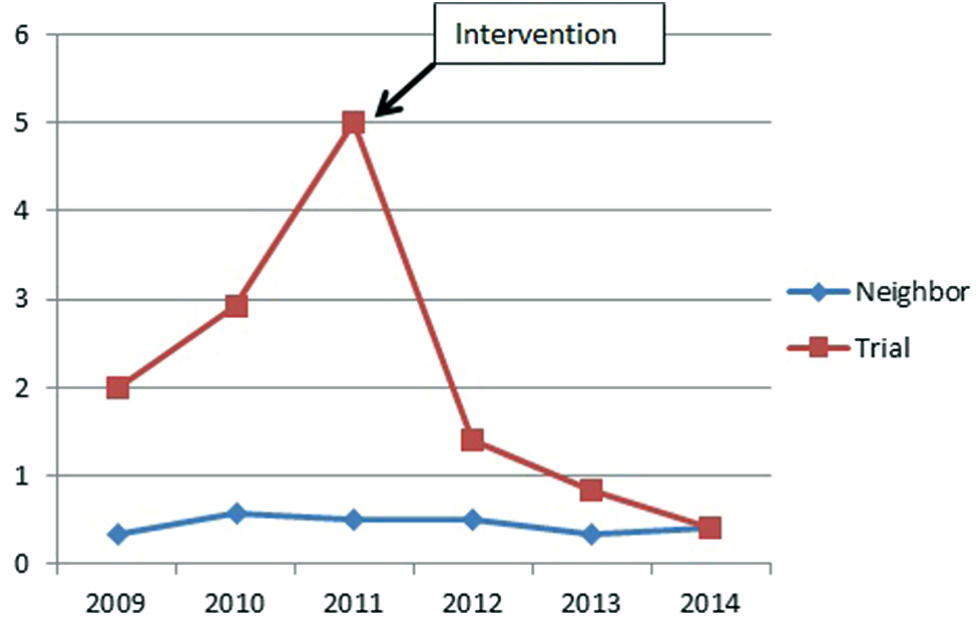
**Yearly force incident rates per 100 club member in 2008–2014, before and after the intervention in spring 2011**.

The yearly force incidence rate in the trial club was nearly halved after the intervention (58%, 95% CI 41–81%), while this rate remained low and stable in a neighboring club. Compared with the neighboring club, the rate in the trial club was four times higher before the trial and reduced to two times higher after trial (rate ratio 4.36, 95% CI 2.41–8.56 and rate ratio 2.28, 95% CI 1.37–3.92, respectively). See also **Figure 2** and **Table 1**.

**Table 1:** Yearly force incident rates per 100 club member in 2008–2014, before and after the intervention in spring 2011.

	Before	After	Rate ratio*	(95% CI)*	
Trial club	27.8	16.0	0.58	0.41	0.81
Neighbor club	6.4	7.0	1.11	0.53	2.40

*Poisson regression.

In the neighboring club, the intervention was introduced in March 2012, giving no changes in yearly force incidence rate after intervention (rate ratio 1.02, 95% CI 0.48–2.13).

### Staff Sick Leave

The average yearly sick leave days was significant reduced in all clubs in the municipality in 2008–2014, but the reduction in the trial club was larger than that of all other clubs (12.0 vs. 5.5 days) and that of a neighbor club (12.0 vs. 8.3 days). See **Table 2**. Compared to all other clubs, the trial club had more sick leave days before the trial and had fewer sick leave days after the trial, with a statistical significant difference of 3.0 days (95% CI 1.8 to 4.2) before trial and of -3.4 days (95% CI -4.3 to -2.6) after trial.

**Table 2:** Average yearly sick leave (days) per employee 2008–2014, before and after the intervention in spring 2011.

	Before	After	Difference	(95% CI)	
Trial	23.3	11.3	−12.0	−13.5	−10.6
Neighbor	26.4	18.1	−8.3	−10.6	−6.0
All other*	20.3	14.7	−5.5	−5.8	−5.2

*Including all other clubs in the municipality.

### Three-Year Follow up Questionnaire

As can be seen from **Table 3**, the response rate to the questions is generally high although varying. In both clubs the coach education and the Resilience Program course is rated higher than the body relaxation course. The Resilience Program is still used by a large majority of the staff in both clubs in communication with the adolescents.

**Table 3:** Staff evaluation of the meaningfulness of three educations, and their specific use of the RP program with adolescents in the club.

	2014 evaluation of Meaningfulness of post-graduate education (10 point scale) average. (xx) = response rate in %			RP use with adolescents
	Coaching 2010–2011	Body relaxation 2009	RP Spring 2011: Trial Late 2012: Neighbor	
Trial club (*N* = 12)	7, 6 (92)	5, 2 (75)	8, 1 (100)	80% (83)
Neighbor club (*N* = 12)	8, 6 (92)	4, 3 (58)	8, 9 (83)	100% (66)

*All members of the staff in the two clubs, except one person who had changed job, filled out the questionnaire.*

Because the scores are not normal distributed, we used Kruskal–Wallis test to check the differences in the distributions of scores between different programs or clubs.

Among trial club, both RP and coaching had a higher score than body relaxation (*p* < 0.05 and *p* < 0.01, respectively), while there was no difference between RP and coaching (*p* = 0.66). The same is true for neighbor club (the corresponding *p*-values: *p* < 0.01, *p* < 0.01, and *p* = 0.57, respectively).

If we compare the same program between trial club and neighbor club, there is no difference for any program (all *p* > 0.10).

## Discussion

Clearly the most interesting result of this study is the fact that the vast majority of the staff is still using the Resilience Program in their daily work 3 years later. The staff themselves rated the Resilience Program as well as the coaching education as very valuable. This impression is confirmed by interviews with the club managers. They consider the simple dissemination of knowledge about mentalization and the neurobiology of mentalization as the key factor in the intervention and this is what is still used by the staff.

Immediately after the intervention and in the following years, the frequency of force incidents and the sick leave decreased in the trial club. There may be two reasons for this development: natural fluctuations in conflict causal factors (regression toward the mean) and/or a positive effect of the intervention. Because this is not a controlled trial, we cannot determine which of the hypotheses is most likely to be true.

Despite the positive evaluation and use of the Resilience Program in the neighbor social club 1 year later in spring 2012, this has not affected the force incident rate in that club. This may indicate that the RP program in the form it was delivered was not effective in relation to this outcome in that club. Another hypothesis may be that the force incident frequency in that club is as low as can be, considering the challenges children and young people and families in this low income area face in their lives. Maybe a further decrease cannot be expected. However, it is also important to notice that the development in the frequency of high risk conflicts leading to incidents of using force in 2009–2010 was very different in the trial club and the neighbor club so one should be cautious in the interpretation of the data since the two clubs in this respect cannot be considered comparable.

## Conclusion

The exploratory pilot study results suggest that the Resilience Program may be promising and that randomzied testing of the program is justified. The program is easy to understand, even for disadvantaged children and adolescents. The results indicate that the program may contribute to building a safe mental environment for disadvantaged adolescents and the staff around them in social youth clubs.

The ongoing randomized trials will show if the Resilience Program is efficient as a completely self-directed online program as well as a group-based education and training program used within organizational contexts such as schools and educational institutions.

May be this type of low cost brief intervention programs focused on education can contribute to the solution of societal mental health problem challenges.

## Conflict of Interest Statement

The authors declare that the research was conducted in the absence of any commercial or financial relationships that could be construed as a potential conflict of interest.

## Footnotes

1 http://myresilience.org

2 ttp://myresilience.org
